# Nephron-sparing surgery for Wilms tumor

**DOI:** 10.3389/fped.2023.1122390

**Published:** 2023-01-20

**Authors:** Andrew J. Murphy, Andrew M. Davidoff

**Affiliations:** ^1^ Department of Surgery, St. Jude Children's Research Hospital, Memphis, TN, United States; ^2^ Division of Pediatric Surgery, Department of Surgery, University of Tennessee Health Science Center, Memphis, TN, United States

**Keywords:** Wilms tumor, nephron-sparing surgery, bilateral, partial nephrectomy, pediatric, kidney

## Abstract

The algorithm that has been used successfully in the surgical management of unilateral Wilms tumor, radical nephroureterectomy, cannot be used in children who present with synchronous bilateral renal masses. Instead, a surgical approach that removes all tumor masses while preserving as much normal renal parenchyma as possible is encouraged to avoid acute and long-term renal insufficiency. We will review technical aspects of the conduct of nephron-sparing surgery for synchronous bilateral Wilms tumor, including the more recent advances in the use of imaging adjuncts such as pre-operative 3D imaging and fluorescence-guided surgery. The potential role of nephron-sparing surgery for unilateral Wilms tumor will also be discussed.

## Introduction

Wilms tumor (WT) is the most common kidney cancer in children (1, 2). Under Children's Oncology Group (COG) protocols, patients with unilateral tumors are typically treated by up-front radical nephroureterectomy with lymph node sampling. In contrast, International Society of Pediatric Oncology (SIOP) protocols recommend neoadjuvant chemotherapy with vincristine and actinomycin followed by radical nephroureterectomy and lymph node sampling (3). While radical nephroureterectomy has been regarded as the worldwide standard of care for unilateral WT in patients without a genetic predisposition, such an approach would render a patient with bilateral WT anephric, and thus nephron-sparing approaches have been developed and refined to preserve long-term kidney function for these patients (4). Nephron-sparing surgery (NSS) is also employed after neoadjuvant chemotherapy in patients with known genetic predisposition to WT, given their elevated risk of metachronous contralateral tumor development (5). Furthermore, there is considerable ongoing debate about the role of NSS after neoadjuvant chemotherapy in anatomically favorable cases of unilateral WT (6).

While bilateral NSS is widely advocated as the optimal surgical approach for patients with bilateral WT, the first multi-institutional treatment study COG AREN0534 showed the rate of bilateral NSS to only be 35% (7). Most patients in this study underwent unilateral radical nephroureterectomy of the more anatomically complicated side and NSS of the more anatomically favorable side. In contrast, our single-institution series from a center specializing in bilateral NSS had a rate of such an approach in greater than 90% of cases (8).

The purpose of the current article is to review the technical aspects and perioperative considerations of NSS for WT. We will also review imaging adjuncts including preoperative three-dimensional imaging and intraoperative fluorescence-guided surgery which may increase the feasibility of a nephron-sparing approach in an individual case. Detailed preoperative and intraoperative anatomic planning and mapping of bilateral WT may increase the chance of successfully performing bilateral NSS. The debatable role for NSS in selected cases of unilateral WT will also be discussed.

### Preoperative evaluation and management

A thorough history and physical examination may suggest a genetic predisposition to WT and therefore warrant an attempt at NSS, even if unilateral. Emphasis should be on detecting aniridia, macroglossia, hemihyperplasia, macrosomia, and genitourinary anomalies, any of which may suggest genetic predisposition to WT (9). If cryptorchidism is present, an inguinal examination and possible MRI can determine the location and character of the testis. For intraabdominal testes, staged orchiopexy may be necessary at the time of abdominal exploration and in a subsequent setting. A recent 5-year unselected cohort of WT patients (unilateral and bilateral) from a single institution demonstrated a 33% rate of detectable germline (epi)genetic predisposition, thus suggesting an expansion of germline genetic testing in WT patients may be warranted (10). Because of the high rate of detectable genetic predisposition in patients with bilateral WT, genetic counseling and possible testing are advised for all patients if resources allow (11).

Biopsy of bilateral renal masses at diagnosis is discouraged by the COG due to the exceedingly low incidence of non-WT bilateral renal masses in children and poor ability to detect anaplastic WT (7, 12). For patients with bilateral renal masses likely to be WT, surgical resection should be conducted at either 6 or 12 weeks after the initiation of induction chemotherapy with vincristine, actinomycin-D, and doxorubicin (7). Longer courses of chemotherapy do not result in meaningful volumetric regression and may promote development of anaplasia in patients with bilateral WT (12). Using this approach, the AREN0534 protocol demonstrated superior outcomes to historical controls who were managed without strict surgical timing guidelines and often received protracted courses of chemotherapy (7). Failure of volumetric regression at six weeks can be due to two common scenarios: treatment resistance due to the presence of anaplasia or rhabdomyoblastic differentiation, the latter of which is particularly relevant in patients with *WT*1 germline pathogenic variants (13). If volumetric regression (defined on COG AREN0534 as 50% reduction in tumor volume according to RECIST 1.1 criteria) is not achieved at six weeks, NSS should be performed if feasible (7, 14). If NSS is not feasible, the AREN0534 protocol advocates for open biopsy at this timepoint to evaluate for anaplasia vs. rhabdomyoblastic differentiation (7).

The decision to perform NSS vs. radical nephroureterectomy is determined by the anatomic characteristics of the tumor in question and the amount of residual normal kidney present on both sides. In general, we advocate for the surgeon to keep an open mind about a nephron-sparing approach until complete tumor and kidney mobilization is performed. Often, a plane of dissection or surgical approach that would not be appreciated on preoperative imaging can be identified in the operating room once the kidney and tumor are completely mobilized and intraoperative ultrasound is performed. For circumstances in which there is minimal or no visible residual normal kidney associated with the tumor in question and there is ample residual normal kidney on the contralateral side, a radical nephroureterectomy should be considered. In contrast, if there is generous residual normal kidney associated with the tumor in question, a nephron-sparing approach should be strongly considered, regardless of the size of the tumor. Even quite large tumors can be resected by a nephron-sparing approach and thus tumor size should not directly determine this decision. It is most often possible to resect tumors using a nephron-sparing approach even when there is vascular abutment or significant displacement. In some cases the benefits of saving renal parenchyma despite a positive margin may outweigh the risks of the radiation therapy that would be required. However, while rare for WT, vascular encasement renders a nephron-sparing approach improbable without local tumor spillage or a positive margin. Therefore, a radical nephroureterectomy should be considered in the circumstance of frank vascular encasement. Extensive invasion of the collecting system is another potential indication for a radical nephroureterectomy; however, it can be quite difficult to differentiate urinary collecting system obstruction due to extrinsic compression from frank invasion based on preoperative imaging. In contrast to the vascular system, partial resection of the collecting system can be accomplished and closed without much morbidity.

### Intraoperative approach and technique

The patient is placed in the supine position on the operating table with the arms tucked or abducted. Two large-bore peripheral IV catheters placed in the upper extremities will typically suffice for intraoperative resuscitation. If such peripheral venous access cannot be obtained, a temporary non-tunneled central venous line can be placed in the internal jugular vein or subclavian vein for resuscitation; however, most patients undergoing NSS will have existing central venous access in place due to prior administration of neoadjuvant chemotherapy. An arterial line is placed for hemodynamic monitoring. It is advised that type and crossmatched blood be available in the operating room given the losses often associated with parenchymal transection of the kidney. An epidural catheter typically provides excellent postoperative pain control in this setting. A Foley catheter is placed to monitor intraoperative and postoperative urine output. Some degree of hematuria is expected during and after the conduct of NSS.

**Figure 1:**
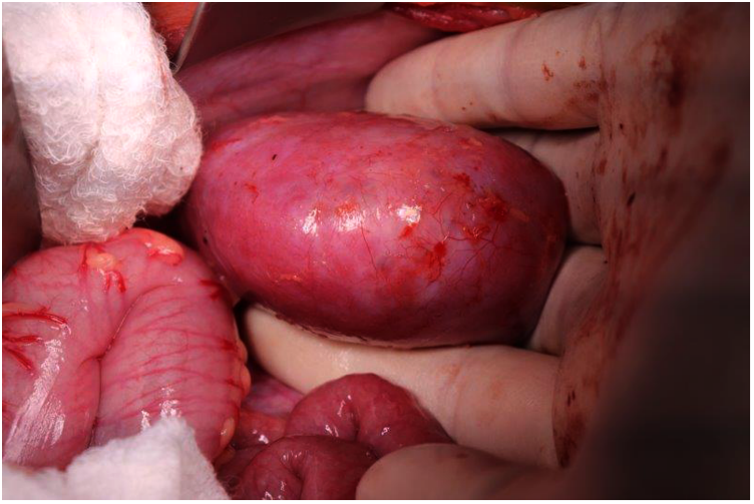
The renal hilum can be accessed and the vessels compressed between the surgeon's fingers after complete mobilization of the kidney/tumor and passing a hand underneath.

**Figure 2:**
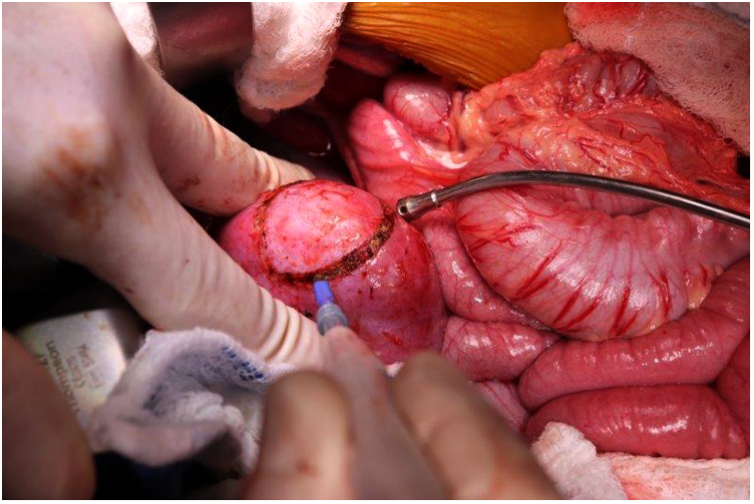
Scoring the renal capsule around the location of the tumor to highlight the anticipated course of dissection.

A bisubcostal laparotomy incision facilitates transabdominal exposure to both kidneys and associated tumors. Alternatively, Lim et al. have described a retroperitoneal approach to tumors using two flank incisions placed midway between the costal margin and iliac crest. This approach was chosen to facilitate bowel function recovery and to isolate potential urine leaks to the retroperitoneum (15). Large tumors may require full medial visceral rotation, while limited mobilization of the colon or colon and duodenum may suffice for smaller tumors. A self-retaining retractor is placed to facilitate exposure. Complete mobilization of the affected kidneys and tumor(s) before initiating division of any kidney parenchyma is critical to the successful conduct of NSS. The ipsilateral adrenal gland can often be spared if there is a plane of dissection between it and the adjacent kidney or tumor capsule (16). Complete mobilization will allow for three-dimensional appreciation of the tumor extent by visual examination, palpation, or intraoperative ultrasound assessment. Furthermore, complete mobilization of the kidney allows access to the vascular hilum of the kidney and enables manual compression of the hilum or parenchyma as needed (Figure 1). Mapping of the kidney dissection plane and scoring the complete intended path of resection along the capsular surface of the kidney are critical to perform before any deeper division or dissection of the kidney parenchyma is undertaken (Figure 2). If the path of resection is not clearly marked at the outset, bleeding during parenchymal transection of the kidney can become disorienting and can lead to deviation from the path most likely to result in complete resection. The main renal vein and artery may be dissected out and encircled with vessel loops to be used for vascular control in the event of significant bleeding. Gentle, deliberate handling of the vascular pedicle is critically important, especially in infants and young children because severe vasospasm or traction injury with resultant vascular thrombosis can occur. During mobilization of the kidney or dissection of the vascular pedicle, if mottling of the kidney parenchyma is noted, a short break with replacement of the kidney into its normal anatomic position can allow transient hypoperfusion to resolve. The vascular pedicle can also be treated with papaverine as needed to address vasospasm. We do not routinely perform vascular clamping or pre-emptively occlude the renal vasculature during parenchymal transection. However, techniques using both cold (vascular clamping with topical cooling or cold intravascular perfusion of the kidney parenchyma) and warm ischemia (vascular clamping without cooling during parenchymal transection) have been described in pediatric NSS with good perioperative outcomes (17–20).

**Figure 3:**
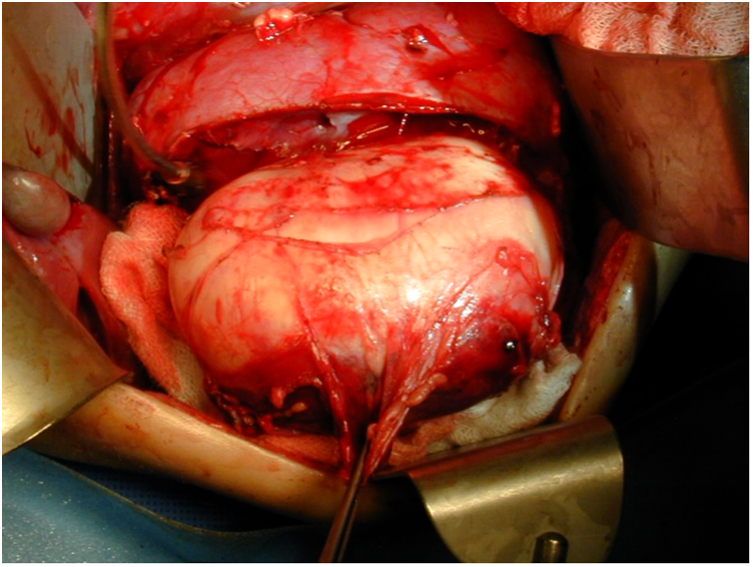
Separating the normal renal parenchyma from the tumor, using an enucleation approach, to ensure maximal parenchyma remains.

**Figure 4:**
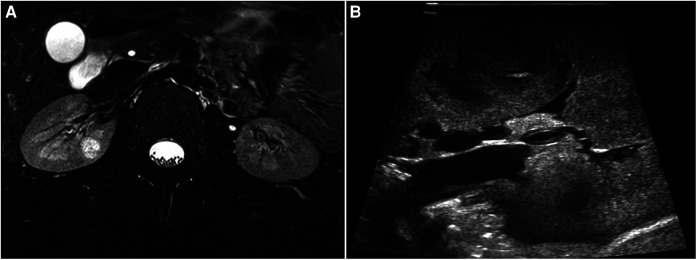
(**A**) Pre-operative MRI of renal lesion within the right kidney parenchyma. (**B**) Lesion localization with intraoperative ultrasound.

For tumor removal, we prefer an enucleation technique (marginal resection) focused on resecting the tumor with an intact capsule to spare the greatest amount of functioning residual renal parenchyma. Unlike tumors that grow with an infiltrative pattern into the adjacent tissues such as sarcomas, WTs grow within a fibrous rim and therefore an enucleation technique typically results in a margin-negative resection. In fact, the presence of a fibrous capsule is the key histologic distinguishing feature between WT and nephrogenic rests. The dissection is performed with a right-angle clamp and electrocautery with intermittent use of a Kitner dissector to develop the appropriate plane of dissection (Figure 3). We have not found intraoperative frozen section to be useful in defining the appropriate plane of dissection or resection margin although others do this routinely. Bleeding during development of this plane of dissection can be temporarily controlled using direct compression with a surgical sponge. All discrete multifocal lesions are addressed in a similar manner during the operation. For small lesions not visible from the capsular surface of the kidney, intraoperative ultrasound is very helpful (Figure 4). We have found that central lesions, even those abutting the central collecting system or renal vasculature can be resected using this technique. If the plane of dissection is initiated at the periphery of the centrally located lesion and dissection works toward the hilum, the lesions are often resectable despite an ominous preoperative imaging appearance. Alternatively, Fuchs et al. have described a longitudinal partial nephrectomy technique for difficult central or hilar lesions in which the kidney is bisected in the longitudinal (coronal) plane starting from the periphery and continued centrally. The central or hilar mass is then enucleated, the collecting system repaired, and the bisected kidney parenchyma reapproximated after the tumor is removed (20, 21). If a partial nephrectomy technique (rather than enucleation) is chosen, extra-parenchymal ligation of vascular branches to the affected pole of the kidney may cause ischemic demarcation of a parenchymal transection path.

**Figure 5:**
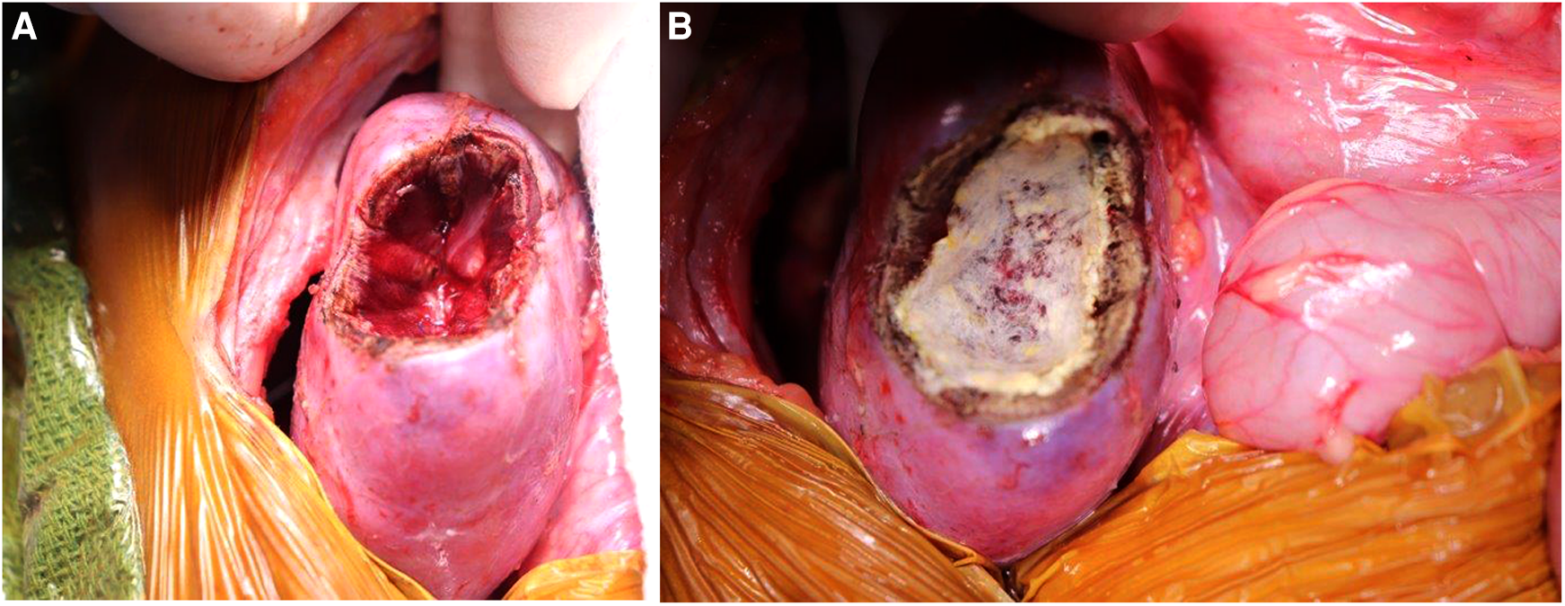
(**A**) Kidney defect following marginal resection of lesion. (**B**) Covering the surface of the defect with TachoSil to ensure hemostasis.

**Figure 6:**
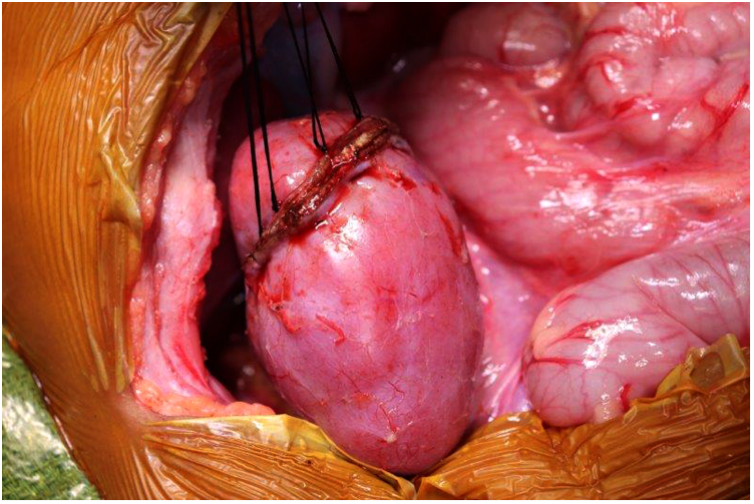
Securing the edges of kidney parenchyma folded over a resection defect with silk mattress sutures.

Once a given lesion is resected, focal areas of arterial or venous bleeding are controlled with figure-of-eight prolene suture. Violations of the collecting system are repaired with running monocryl suture. Topical anticoagulants such as thrombin-soaked gelfoam followed by TachoSil fibrin sealant patch facilitate hemostasis on the cut surface of the kidney (Figure 5) (22). If the residual renal parenchyma can be closed over the resection bed in a folded manner without undue tension, we secure it in this position with silk horizontal mattress suture (Figure 6). We do not routinely place ureteral stents or peritoneal drains unless there is significant concern about tension or complexity of the collecting system closure. Despite total mobilization of the kidney during surgery, we do not place pexy sutures to the retroperitoneum at the conclusion of the case and have not experienced torsion of the kidney following such a procedure.

Intravascular thrombus is not a strict contraindication to NSS; however, successful NSS in such a situation is dependent on venous thrombus regression with neoadjuvant therapy or the presence of a branched or accessory renal venous system in which venous drainage can be preserved to the renal remnant (23). A staged operation in which each side is addressed in a separate operative setting may be preferred in such cases. A staged approach ensures successful preservation of a functioning renal remnant before the contralateral side is manipulated; however, the tradeoff of such an approach is a potential delay in chemotherapy. Radical nephroureterectomy on the side with venous thrombus is likely the safer approach in cases of extensive thrombus or suprahepatic involvement.

SIOP has developed a standardized NSS “formula” that includes documentation of the surgical technique, intraoperative assessment of the surgical resection margin, the pathologic resection margin, and the estimated percentage of remaining renal parenchyma in each kidney at the conclusion of the procedure (Table 1) (24). Mrad et al. demonstrated that patients with an estimated remaining renal parenchyma percentage of less than 50% had hypertension and detection of proteinuria at a median of eight-year follow-up (25). Such synoptic reporting of operative features may help standardize data reporting for comparison of oncologic and long-term renal function outcomes.

**Table 1:** International Society of Pediatric Oncology (SIOP) standardized reporting “formula” for nephron sparing surgery.

Reporting Component Format: *NSS (X)-SRM (n)-PRM (n)-RRP (n%)*	Description
1. Surgical Technique	
a. NSS (A)—partial nephrectomy	Resection of the tumor with a rim of normal renal parenchyma
b. NSS (B)—enucleation (marginal resection)	Resection of the tumor without a rim of normal renal parenchyma
2. Surgical Resection Margin (SRM)	Surgeon's impression of resection margin
a. Intact pseudo-capsule = (0)	
b. Doubt intact pseudo-capsule = (1)	
c. Definite tumor breach = (2)	
3. Pathological Resection Margin (PRM)	Microscopic resection margin on permanent pathology
a. Rim of normal renal parenchyma on resection margin (=0)	Exception for nephroblastomatosis
b. Intact pseudo-capsule along resection margin (=1)	
c. Tumor breach (=2)	
4. Remaining Renal Parenchyma (RRP) = (*n*%)	Surgeon's assessment of the percentage of remaining normal renal parenchyma

NSS, nephron-sparing surgery; SRM, surgical resection margin; PRM, pathological resection margin; RRP, remaining renal parenchyma. Table is adapted from reference (23).

Lymph node sampling is regarded as a mandatory endeavor during WT surgery to assign the appropriate local disease stage to the tumor to guide adjuvant therapy. Paracaval, paraaortic, aortocaval, and parahilar lymph nodes are sampled for local staging on each side.

### Repeat nephron-sparing surgery

Repeat NSS may be necessary due to missed small lesions, local relapse, or metachronous tumor development. Although adhesiolysis, renal mobilization, and identification of the tumor boundaries may be more difficult due to the reoperative field, the procedure is feasible with good renal function outcomes (26). Detection of diffuse anaplasia or blastemal predominance in reoperative NSS specimens is associated with poor outcomes and may prompt multidisciplinary discussion about the role of completion nephrectomy in that setting (26). Diffuse anaplasia is associated with poor outcomes in patients with bilateral WT. Therefore, our practice is to perform completion nephrectomy if diffuse anaplasia is detected in tumors with local stage III disease due to a positive margin. In contrast, we do not perform completion nephrectomy if diffuse anaplasia is detected in a tumor resected by NSS with a negative margin (local stage I or II disease).

### Imaging adjuncts

**Figure 7:**
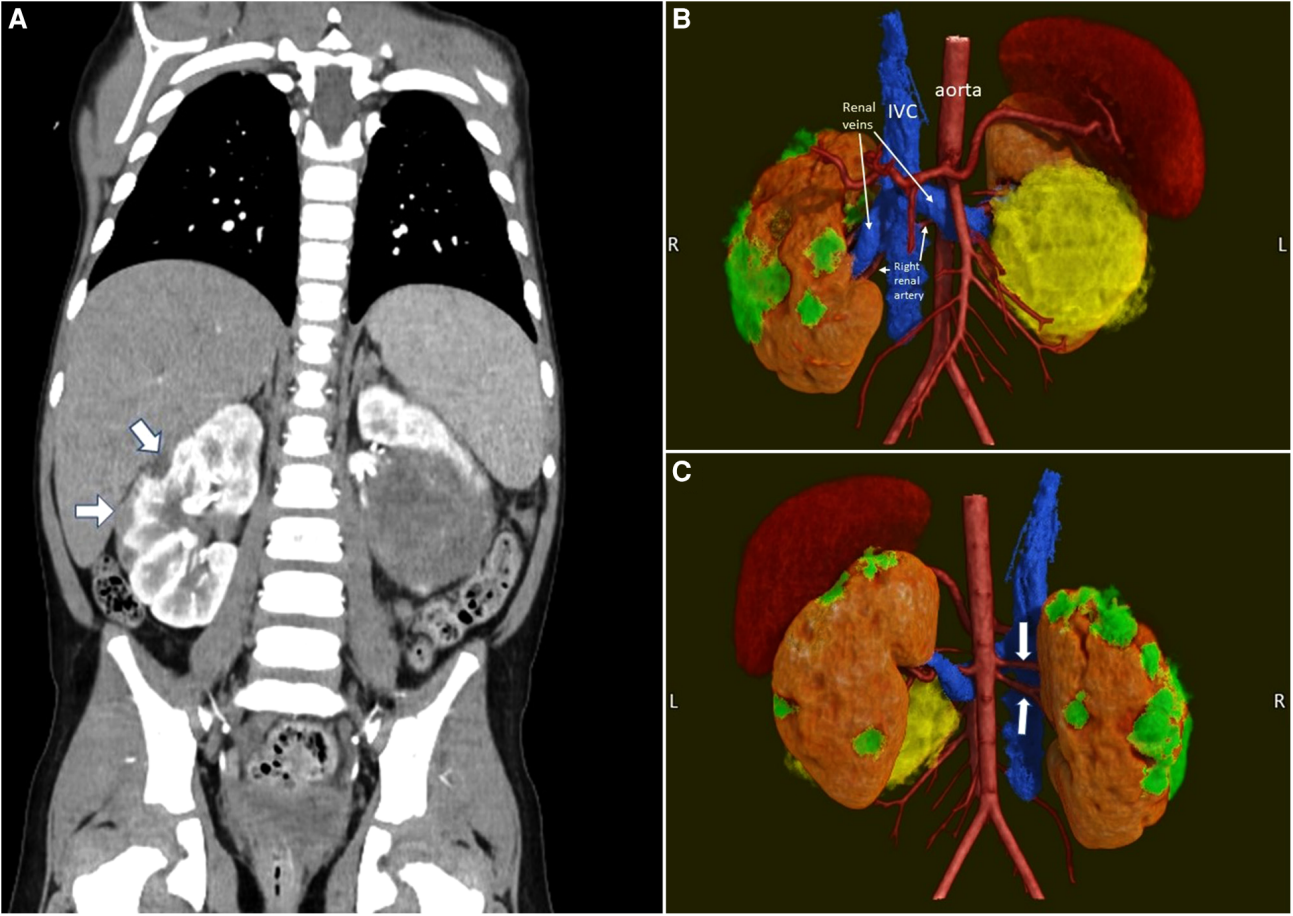
Pre-operative imaging of a case of synchronous bilateral Wilms tumor. (**A**) Pre-operative CT scan. Note the multiple peripheral lesions on the right kidney. (**B**) 3D rendering (anterior view) of the kidneys, their tumors and the vasculature. The smaller right kidney tumors are in green. The large left kidney tumor is in yellow. (**C**) 3D rendering (posterior view). Note the two right renal arteries (white arrows).

Preoperative cross-sectional imaging to assesses the renal arterial and venous anatomy is essential to the planning of NSS. Furthermore, assessment of the tumor relationship to the collecting system is important in NSS. The number, branching pattern, and anatomic relationship of each renal vein or artery to the tumor(s) is important to understand in three-dimensions prior to the conduct of the operation. A dual-phase CT abdomen/pelvis with delayed views to capture the collecting system or MRI scan typically obtain this needed information. Diffusion-weighted imaging MRI may be able to estimate the cellularity of WTs and differentiate tumors with blastemal vs. stromal or skeletal muscle differentiation (27). This could guide decision-making regarding the timing of NSS in the context of tumor volumetric regression and is the subject of considerable future interest. We have found three-dimensional reconstruction particularly useful in facilitating the planning of NSS (Figure 7). Three dimensional reconstructions can be used to generate 3D printed models of the tumor and renal anatomy or the reconstructions can be viewed using virtual reality systems to enhance appreciation of tumor anatomy (28–30). Van der Zee et al. have described a “virtual resection” in which real time manipulation of 3D reconstructions is utilized to plan NSS and to estimate the expected residual renal volume after resection (31). Preoperative nephrometry scoring has been shown to correlate with the feasibility and outcome of a nephron-sparing approach in adult renal tumors (32, 33). This has not been evaluated previously in bilateral WT, but is the subject of ongoing investigation.

A nuclear medicine split renal function scan can estimate the relative contributions of each kidney to overall renal function and can be informative for operative decision making in which an extremely small renal remnant may be predicted after NSS. In such cases, a split renal function scan indicating the involved kidney is not contributing much to renal function may suggest a radical nephroureterectomy should be performed on that side. However, one must also consider that extrinsic compression of the kidney and/or obstruction of the collecting system by a large tumor can cause reversible impairment of kidney function. Therefore, NSS can still be considered if it is anatomically feasible, even when the adjacent non-diseased kidney has decreased contribution to renal function as assessed by the preoperative scan.

Fluorescence-guided surgical approaches may assist in the differentiation of normal kidney from tumor tissue, particularly in cases with multifocal tumors present in each kidney (34). Interestingly, a recent report showed that 1.5 mg/kg indocyanine green administered the day prior to surgery resulted in intraoperative fluorescence of the normal kidney and a paucity of fluorescence in tumor tissue during bilateral NSS for WT (35). This finding contrasts with most pediatric solid tumors, in which the tumor tends to fluoresce relative to the adjacent normal tissue because of the enhanced permeability effect (increased uptake and delayed excretion of indocyanine green characteristic of most tumor masses) (34). Intraoperative injection of ICG into the perihilar lymphatics or adjacent normal kidney is a technique being evaluated to identify draining lymph nodes and facilitate lymph node sampling (36). In the future, monoclonal antibody-based fluorescent probes and/or activatable fluorescent probes may increase the tumor histology specificity of such approaches and can be tailored to tumor-specific cell-surface biomarkers (37).

### Postoperative management and complications

The local stage of each tumor should be assessed and documented by the surgeon and the pathologist. Radiographic evidence of preoperative rupture (free fluid in the pelvis or retroperitoneum with tumor surface irregularities) and any intraoperative confirmation should be documented by the surgeon. Because neoadjuvant chemotherapy is administered to nearly all patients undergoing NSS for WT, there is unlikely to be residual pelvic free fluid from a preoperative rupture at the time of the operation. However, soft tissue discoloration or adhesions may corroborate rupture indicated on preoperative imaging. Any intraoperative spill of tumor contents (and the extent of such spill) or gross residual positive margins should be documented by the surgeon. The microscopic margins of resection and any lymph node positivity should be documented by the pathologist. The SIOP post-treatment histology risk is assessed according to the percentage of necrosis, blastema, and any diffuse anaplasia present in each tumor specimen (Table 2) (38). A combination of local disease stage and post-treatment histology will guide adjuvant therapy selection (4).

**Table 2:** Post chemotherapy histology risk group assignment according to SIOP^a^.

Percent necrosis	Histology	Risk group
100% (completely necrotic)^b^	Necrotic	Low
>67% (regressive)	>67% any favorable (i.e., epithelial, stromal, blastemal, mixed)	Intermediate
<67%	>67% Epithelial	Intermediate
<67%	>67% Stromal	Intermediate
<67%	Mixed	Intermediate
<67%	>67% (blastemal predominant)	High
Any	Diffuse anaplasia	High

^a^ Risk-group assigned according to the highest-risk Wilms tumor in each patient.

^b^ Allows for presence of residual, viable nephrogenic rests.

Perioperative complications are significantly higher in NSS than in radical nephroureterectomy, including intraoperative blood loss and postoperative urine leak (39). Postoperative urine leaks from the collecting system can typically be managed non-operatively with transperitoneal drainage, continued Foley catheter use, and cystoscopic placement of ureteral stents.

The rate of microscopic positive margins is significantly higher in nephron-sparing approaches than in radical nephroureterectomy (39). A positive margin will necessitate adjuvant flank radiotherapy on the affected side. Positive margins were present in 36% of kidneys in patients who underwent NSS at our institution (39). However, with appropriate utilization of adjuvant chemotherapy and radiation, local recurrence rates and overall survival are excellent even in patients with positive microscopic margins (7, 8).

### Long-term outcomes

Long-term renal function outcomes are generally favorable after bilateral NSS. In our single institution series of 42 patients treated for synchronous bilateral WT, no surviving patients had a glomerular filtration rate less than 60 ml/min/1.73 m^2^ at a median follow-up of 4.1 years (8). However, a comprehensive renal function evaluation in this patient cohort demonstrated subtle detectable renal dysfunction indicated by microalbuminuria, elevated serum creatinine, or microglobulinuria in 39.3% of patients who underwent evaluation (40). Furthermore, 1/3 of the cohort were taking antihypertensive medications at the time of last evaluation. These features suggest an evolving, clinically relevant picture of renal dysfunction and potentially hypertension-related cardiovascular disease that must be surveyed and treated in patients who undergo bilateral NSS. Long-term renal function outcomes in the cohort treated on COG AREN0534 are being prospectively followed but have not yet been reported. A discussion of detailed renal function surveillance in patients treated for bilateral WT is beyond the scope of this article.

### Nephron-sparing surgery for unilateral Wilms tumor

NSS is recommended in patients with unilateral WT and genetic predisposition due to the risk of metachronous contralateral tumor development and/or the long-term risk of renal failure. The most common mechanisms of WT predisposition include 11p15-related WT (Beckwith-Wiedemann spectrum), *WT*1 disorder, *REST*-related WT, *TRIM*28-related WT, and WAGR (Wilms tumor, aniridia, genitourinary anomalies, range of developmental delays) spectrum (Table 3). Comprehensive reviews of all known genetic and epigenetic alterations that predispose to WT have recently been published (9, 41). Although radical nephroureterectomy is currently regarded as the worldwide standard of care for unilateral WT in patients without known genetic predisposition, there has been increased interest and utilization of nephron-sparing approaches in patients with anatomically favorable unilateral WTs after the administration of neoadjuvant chemotherapy.

**Table 3:** Most common syndromes and pathogenic germline (Epi)genetic variants associated with wilms tumor predisposition.

Syndrome	Germline Pathogenic (Epi)genetic Variants	Clinical Manifestations	Risk of Wilms tumor
Beckwith-Wiedemann Spectrum	11p15.5 site-specific gain of methylation H19/ICR1 11p15.5 loss of heterozygosity (copy neutral loss of heterozygosity) 11p15.5 ICR2 loss of methylation Others	Macrosomia/visceromegaly Macroglossia Hemihyperplasia Omphalocele/abdominal wall defects Ear creases/pits Adrenocortical cytomegaly Kidney abnormalities Risk for embryonal tumors (Wilms tumor, hepatoblastoma, embryonal rhabdomyosarcoma, neuroblastoma, adrenocortical carcinoma)	Varies according to type of (epi)genetic variant: 7–25% overall Greatest risk: 11p15.5 site specific gain of methylation H19/ICR1 Lowest risk: 11p15.5 ICR2 loss of methylation
*WT*1 disorder	Pathogenic variants in *WT*1 (11p13.3) Truncating variants Exon 8/9 missense variants Intron 9 variants Others	Heterogeneous spectrum of: Genitourinary anomalies Disorders of sexual development Steroid-resistant nephrotic syndrome Renal mesangial sclerosis Gonadoblastoma	Varies according to type of genetic variant: Truncating WT1 variants: >80% Exon 8/9 missense variants: 70%–80% Intron 9 variants: 2%
WAGR spectrum	Heterozygous germline deletion 11p13.3 involving contiguous genes *WT*1 and *PAX*6	Wilms tumor Aniridia Genitourinary anomalies Range of developmental impairments	40%–60%
*REST*-related Wilms tumor	Pathogenic variants in *REST*	No consistent phenotype other than Wilms tumor predisposition	Unknown
*TRIM*28-related Wilms tumor	*TRIM*28 truncating or splice site pathogenic germline variants; Maternal parent-of origin effect	No consistent phenotype other than Wilms tumor predisposition Epithelial-predominant Wilms tumor	Unknown

In unilateral WT patients without genetic predisposition, the technical feasibility and potential benefits of a nephron-sparing approach must be weighed against the increased risk of intraoperative tumor spill or a positive margin in NSS, either of which result in local stage III disease and require three-drug chemotherapy and flank irradiation. The other important consideration is the questionable benefit of this approach given the limited rates of long-term renal failure in unilateral WT patients without genetic predisposition who underwent radical nephroureterectomy (42). Considering this important risk-benefit comparison, the SIOP-2001 protocol allowed for a nephron-sparing approach to be performed for polar or peripheral, non-infiltrating WTs after neoadjuvant chemotherapy in which a clear margin could be anticipated. The open SIOP-RTSG Umbrella protocol allows for NSS in unilateral non-syndromic WT in cases in which the tumor is polar or peripheral, <300 ml in volume, without evidence of preoperative rupture, no intraluminal renal pelvic tumor, no adjacent organ invasion, no intravascular thrombus, no multifocality, and in which a negative margin with a renal remnant having greater than 66% of the original kidney volume is expected (6). NSS is generally not advised for up-front resection of suspected WT; with tiny lesions detected in patients undergoing screening for renal tumors perhaps being a notable, infrequent exception. This makes it much less likely that NSS would be performed in COG institutions than SIOP ones. It is also generally advised that NSS for WT be performed using an open rather than minimally invasive approach, and by a surgeon experienced with this technique.

### Minimally invasive nephron-sparing surgery for Wilms tumor

Although an open approach is generally advocated for NSS in WT due to the concerns noted above, improvements in technology and surgical experience may render minimally invasive NSS feasible in carefully selected patients with anatomically favorable tumors after neoadjuvant chemotherapy. Sala et al. described a case of robotic-assisted unilateral radical nephroureterectomy and contralateral nephron-sparing surgery performed for a patient with bilateral WT after neoadjuvant chemotherapy (43). Lopes et al. reported a series of six patients with unilateral WT for whom a hybrid approach was utilized: the procedure was started by laparoscopic exploration, lymph node sampling, adrenal-sparing kidney mobilization, and vascular isolation/control and then NSS was performed through a small flank incision (44). One of these six patients underwent radical nephroureterectomy because of inability to define an approach likely to achieve a negative surgical margin and all patients in the series ultimately had negative surgical margins (44).

## Conclusions

Bilateral NSS can be performed in most patients with synchronous bilateral WT after neoadjuvant chemotherapy. Successful bilateral NSS is dependent on detailed pre-operative and intraoperative planning, which may be facilitated by 3D reconstruction or 3D printing of preoperative images. Despite a higher rate of positive margins and more postoperative complications when compared to radical nephroureterectomy, oncologic and long-term renal function outcomes are excellent with NSS. Additional long-term follow-up will be required to better understand how renal function is impacted as these survivors age. The ongoing debate about NSS for unilateral WT must balance the theoretical advantages of renal parenchymal preservation against the extremely favorable long-term renal function outcomes seen in unilateral WT survivors who undergo radical nephroureterectomy. Furthermore, this debate must continue to consider the oncologic consequences of positive margins or intraoperative tumor spill that require treatment intensification and its long-term accompanied toxicities.

## Author contributions

AM and AD: contributed to the conception and design of this review article. AM and AD: wrote, edited, and approved the submitted version of the manuscript. All authors contributed to the article and approved the submitted version.
